# Trapped and traumatised: a scoping review of the psychological sequelae of entrapment following motor vehicle collision

**DOI:** 10.1186/s13049-026-01558-9

**Published:** 2026-01-29

**Authors:** Thomas A. M. Dixon, Kara Hole, Louise Johnson, Tim Nutbeam

**Affiliations:** 1 University Hospitals Plymouth, Plymouth, UK; 2 Torbay and South Devon NHS Foundation Trust, Torbay, UK; 3 University of Plymouth Peninsula Medical School, Plymouth, UK; 4 Leeds Major Trauma Centre, Leeds Teaching Hospitals NHS Trust, Leeds General Infirmary, Leeds, UK; 5 IMPACT; Centre for Post-Collision Research, Innovation and Translation, Exeter, UK

**Keywords:** MVC, Motor Vehicle Collision, Entrapment, Mental health, PTSD, Acute Stress, Depression, Anxiety

## Abstract

**Background:**

Motor vehicle collisions (MVCs) are a significant public health challenge, resulting in substantial mortality and morbidity. Entrapment occurs in 12–33% of all MVCs and is associated with greater injury severity, longer on-scene time, and increased mortality. However, the psychological consequences of entrapment remain poorly characterised.

**Objectives:**

This scoping review seeks to establish the current understanding of the psychological sequelae of entrapment following MVCs and draws on insights from related fields of study in order to inform future research and clinical practice.

**Methods:**

A scoping review was undertaken following established methodological guidance. Searches were completed on NHS Knowledge and Library Hub, Proquest, and Ovid. Eligible studies reporting psychological outcomes in casualties trapped following MVCs were included, with no restrictions to publication date. Secondary searches were also undertaken to explore psychological outcomes in the related contexts of MVCs more generally and entrapment in non-MVC settings, with salient findings presented to contextualise the primary search within more established literature.

**Results:**

Seven studies were included from the primary search. One additional study from the secondary searches was included in the primary research discussion due to a high degree of specificity to that topic. No study directly compared psychological outcomes in trapped versus non-trapped casualties. Reported experiences highlighted themes such as perceived threat to life, loss of control and the importance of supportive human contact. Some individuals experienced clinically significant mental health outcomes including post-traumatic stress disorder (PTSD), Acute Stress Disorder (ASD), anxiety, and depression. Secondary evidence supports the likelihood of increased psychological morbidity associated with MVC-entrapment.

**Conclusion:**

Direct evidence on the psychological sequelae of MVC-entrapment is limited. Available findings suggest entrapment may heighten psychological risk through a range of factors, but prospective cohort studies comparing trapped vs non-trapped MVC casualties are needed to determine causal pathways. Further research should also explore rescue-based interventions such as facilitated self-extrication and deployment of extrication buddies, as well as appropriate trauma-informed psychological interventions in the immediate hospital setting and beyond.

**Supplementary Information:**

The online version contains supplementary material available at 10.1186/s13049-026-01558-9.

**Table:** 

Definitions
**Entrapment** | The inability to autonomously escape due to physical restriction, medical causes, or both.
**MVC(s),** **generally/MVC(s) in general** | All forms of motor vehicle collision where no form of entrapment is described.
**MVC-entrapment** | Motor vehicle collisions resulting in entrapment of casualties.
**Non-MVC entrapment** | Any form of entrapment not related to motor vehicle collisions.

## Background

Motor vehicle collisions (MVCs) are a major global health concern, resulting in substantial mortality, and morbidity [1]. Psychological distress following all forms of motor vehicle collision (MVCs, generally), is well documented with survivors experiencing elevated rates of post-traumatic stress disorder (PTSD), acute stress disorder (ASD), anxiety, depression and other trauma-related difficulties [2–5]. Estimates from meta-analyses suggest a PTSD prevalence of approximately 22% among adult survivors, with similarly elevated rates of ASD and other psychological sequelae also reported [2–5]. These difficulties may persist long after physical recovery and can affect return to work, social functioning, and quality of life [6, 7].

Within this wider population, individuals who are unable to exit their vehicle without assistance represent a distinct subgroup of MVC survivors. Entrapment is relatively common (12–33%) in MVCs requiring emergency service response (based on data from England and Brazil) [8, 9] and is associated with greater injury severity, longer on-scene time, and increased mortality [10]. In addition to physical injury, entrapment following MVCs (MVC-entrapment) can involve prolonged exposure to pain, fear, the inability to escape and a perceived threat to life [11]. These peri-traumatic features are recognised contributors to psychological distress in a range of trauma contexts [12–17]. Despite the plausible relevance of these mechanisms, the specific psychological sequelae of MVC-entrapment remain poorly characterised.

No comprehensive synthesis has examined the psychological outcomes specific to survivors of MVC-entrapment. While a substantial body of literature describes psychological outcomes following MVCs in general, this evidence rarely distinguishes trapped from non-trapped casualties, limiting its ability to inform whether entrapment confers additional psychological risk [4, 12, 13]. However, research conducted in non-MVC contexts where entrapment occurs (non-MVC entrapment)—such as natural disasters, mining incidents, and collapsed structures—has identified associations between entrapment and increased risk of PTSD, ASD, anxiety and depression [16–22]. These findings may offer relevant insights due to shared psychological features, including perceived threat to life and loss of control, although differences in context and mechanism mean conclusions cannot be assumed to transfer directly.

Given the absence of direct evidence there is value in mapping existing knowledge in this area. Understanding how entrapment following MVCs may influence psychological outcomes has potential implications for both clinical and emergency response practice, including the provision of reassurance and communication during extrication, early assessment, and follow-up support.

To explore this topic, a scoping review methodology was selected to enable the inclusion of varied study designs and allow exploration of related evidence in an area where direct research remains limited.

The aims of this scoping review are to:

(i) identify and synthesise existing evidence regarding psychological sequelae associated with entrapment following MVCs;
(ii) draw on relevant evidence from adjacent fields where entrapment occurs in order to contextualise potential psychological risk;
(iii) highlight gaps in the current literature to guide future research and clinical priorities.

## Methods

### Overview

The initial objective of this scoping review was to explore the psychological and mental health outcomes experienced by individuals trapped as a result of MVCs. A preliminary search was conducted to identify literature relevant to this focus. However, this search revealed a substantial gap in the existing body of evidence, with no studies addressing the primary research question, empirically.

**Fig. 1:**
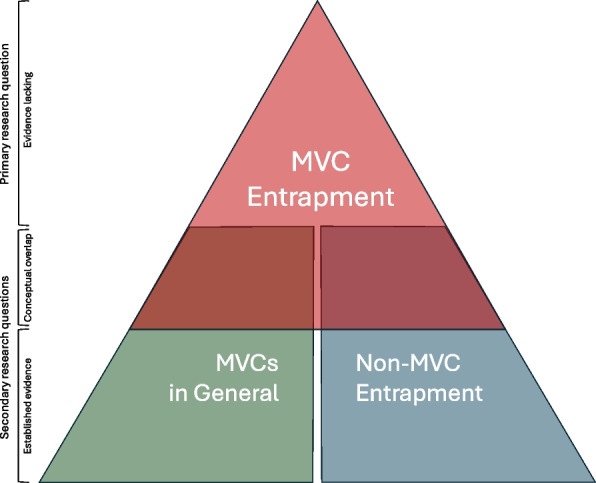
Schematic diagram representing the available evidence relating to both the primary research question, secondary searches and their conceptual overlap

In response, and in line with the iterative approach of scoping review methodology, we repeated our search strategy for two supplementary areas which overlap conceptually with the primary topic (Fig. 1.).

This approach allowed us to methodically explore adjacent bodies of literature, illuminating insights such as prevalence statistics to inform hypotheses regarding the types and degrees of psychological harms MVC-entrapment survivors may sustain.

### Registration and guidance

A protocol for this review was not preregistered, reflecting the iterative nature of scoping review methodology and the absence of a sufficiently defined evidence base at project initiation. All stages followed recognised scoping review guidance, including the methodological framework proposed by Arksey and O’Malley [23] with subsequent refinements by Levac et al. [24], and informed by the Joanna Briggs Institute (JBI) guidance on scoping review conduct [25]. These frameworks support an iterative search process, the inclusion of diverse study designs, and the mapping of existing knowledge and evidence gaps, which align with the objectives of the present review. This scoping review adhered to the PRISMA-ScR checklist [26] wherever practicable, with minor deviations (primarily due to the qualitative nature of the data) noted in Appendix 6 for full transparency.

### Search strategy

A literature review was completed in January 2024 with professional assistance from the South Devon Health Library Service. Database searches took place on the following platforms: NHS Knowledge and Library Hub discovery system, Proquest (PsycArticles, PsycInfo, British Nursing Index, Health Research Premium Collection, PTSDPubs), and Ovid (Medline and Embase). Appropriate search terms were developed to address our question, where relevant natural language and controlled vocabulary terms were selected and combined (for a complete list of all search terms used, see Appendix 1). In the Knowledge & Library Hub search expanders (include related words, full-text searches, equivalent subjects) were applied to improve the sensitivity of search results. Limiters were applied where necessary (peer reviewed, subject headings) to improve precision. No limitations were applied to the publication date for our primary search.

### Eligibility criteria, screening and selection of sources

After removing duplicates, 153 articles were identified. Two authors independently screened titles and then full texts using predefined criteria: Results were considered eligible if they were available in English and discussed the psychological outcomes for patients experiencing entrapment as a result of a MVC of any kind. Both qualitative and quantitative studies were included. Articles focusing solely on the psychological consequences for rescue teams, rather than casualties themselves were excluded, as were those with no specific mention of entrapment. No disagreements occurred; if they had, a third reviewer would have been recruited to repeat the same process and reach consensus.

### Data extraction and synthesis

Two reviewers identified relevant information from each included study, extracting key data and themes (e.g., population, psychological outcomes, experiences of entrapment) into summary tables. Synthesis was completed by grouping the extracted findings into prominent themes and discussing these in a narrative format.

### Secondary searches

As described above, two secondary searches were undertaken to provide contextual background and position the primary findings within the wider literature. These encompassed:

A. Mental health outcomes following all MVCs, regardless of entrapment status (Appendix 2).
B. Mental health outcomes associated with entrapment in non-MVC contexts (e.g., natural disasters, mining incidents) (Appendix 3).

Prevalence findings from the secondary searches were used to provide conceptual context rather than being incorporated into the primary thematic synthesis, with the exception of one study which specifically describes entrapment following a bus crash and was therefore moved to the primary research discussion. Although risk factors were also identified, their scope and complexity warrant separate analysis. The secondary searches followed the same methodology and inclusion criteria as the primary search (adjusted for the secondary contexts of MVCs, generally and non-MVC entrapment), with a 20-year publication limiter applied due to the volume of available literature. To maintain relevance, we reported only the highest-level evidence (meta-analyses, systematic reviews, and larger cohort studies). There is no crossover between review articles and individual studies discussed in this paper. Summary tables for all identified studies—including smaller or context-specific papers not discussed in the main text—are available in the supplementary materials (Appendices 4 and 5).

## Results

Our primary search identified scarce literature on the psychological impacts of MVC-entrapment, and no study directly addressed the review question. After title and full-text screening, seven papers met the eligibility criteria, with one additional study identified through secondary searches included due to its direct relevance to casualty experience during MVC-entrapment. There were no randomised controlled trials, no cohort studies, no intervention arms, and the included papers sit at the lower end of the evidence hierarchy (essentially case reports and case series).

**Fig. 2:**
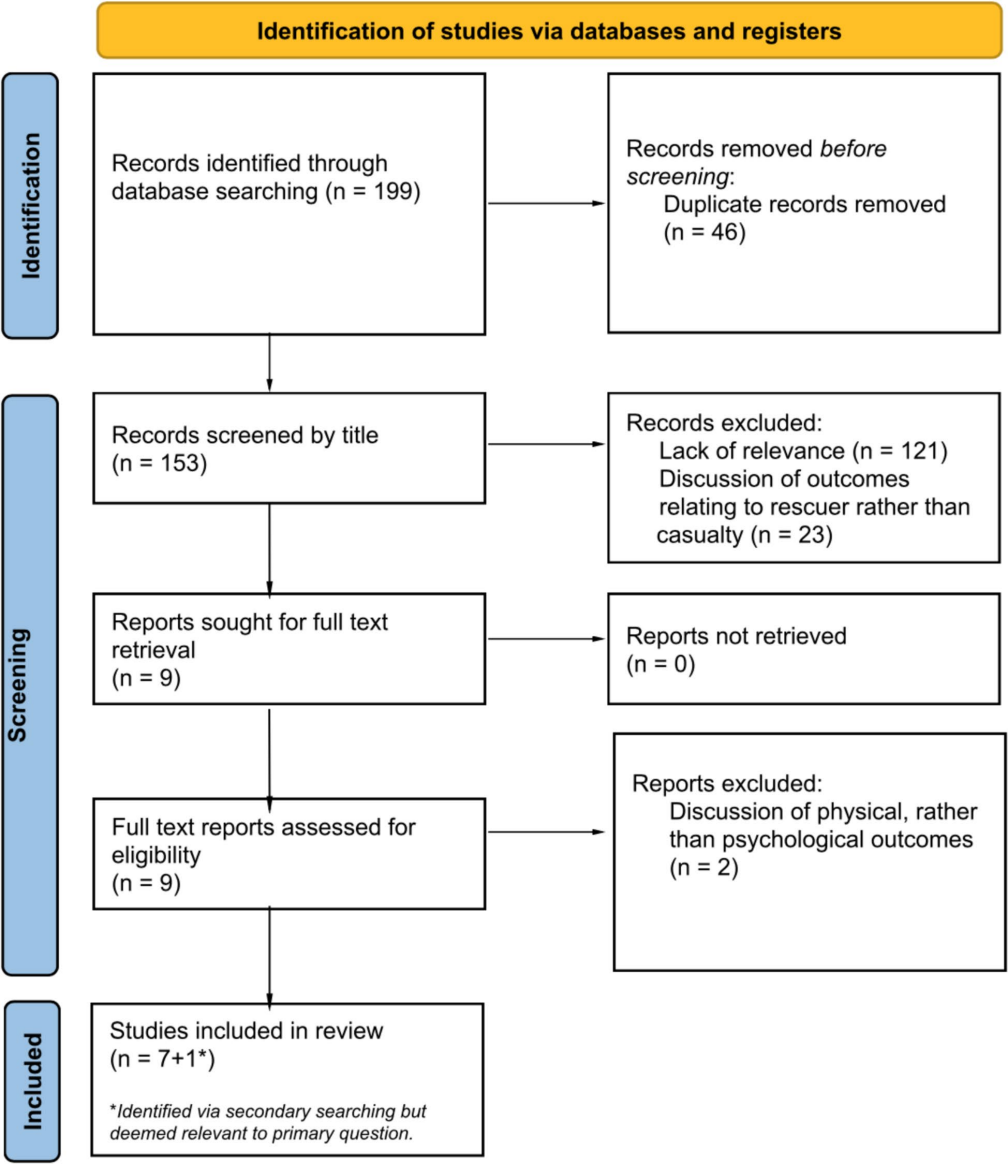
PRISMA flow-chart outlining the study selection process for the primary search [27]

The full selection process is presented in the PRISMA diagram (Fig. 2), and key characteristics and findings from the eight included studies are summarised in Table 1.

**Table 1:** Summary of papers selected for full text review

Author	Year published	Title	Article Type	Vehicle Type	Participants	Degree of Entrapment	Psychological Outcomes Reported	Key Themes
Secklin, P. L [30]	2001	Multiple Fractures in Time: Reflections on a Car Crash	Autobiographical essay	Car	One	Experienced full MVC entrapment	**Some features of DSM-5-TR diagnostic criteria for PTSD**, nightmares, absence of key memories	Pain, fear, perceived threat to life, memory disturbance, physical/mental incapacitation, value of supportive human contact
Lanius, R. A., Hopper, J. W. & Menon, R. S [32]	2003	Individual differences in a husband and wife who developed PTSD after a motor vehicle accident: A functional MRI case study	Dual case study	Car	Two (same- vehicle)	Both experienced full MVC entrapment in multi-vehicle MVC; self/co-extricated	**Clinical diagnoses of ASD and PTSD,** flashbacks, nightmares, psychological & physiological arousal, avoidance, poor sleep, poor concentration, startle reactions, irritability, feelings of numbness	Clinically significant mental health outcomes, fear, witnessing death, perceived threat to life, loss of control or restricted ability to act, active versus passive role during extrication
De-Soir, E. & Goffings, K [33]	2011	Psychological stabilisation for MVA victims	Pilot study	Not stated	Six (separate- MVCs)	All experienced full MVC entrapment requiring extrication by emergency responders	No specific psychological outcomes reported	Sense of powerlessness, value of supportive human contact, value of understanding/being involved in extrication plans
Cowley, A [31]	2014	Self-extrication in road traffic collisions: do we really need to cut the roof off?	Literature review	-	-	-	Increasing anxiety during entrapment	Physical discomfort, pain, anxiety
Farago, M. A. & Pop, M. S [34]	2018	The management of musculoskeletal pain and disfunction from traffic accidents polytrauma	Dual case study	Car	Two (separate- MVCs)	Both experienced full MVC entrapment requiring extrication by emergency responders	**Clinical diagnoses of ASD and PTSD,** symptoms of anxiety and depression, amnesia, negative appraisals, intrusive memories, aggression, irritability, poor attention, emotional- lability, startle responses	Clinically significant mental health outcomes, pain, anxiety, low mood, memory disturbance, intrusive memories
Nutbeam, T., Brandling, J., Wallis, L. A. & Stassen, W [11]	2022	Understanding people’s experiences of extrication while being trapped in motor vehicles: a qualitative interview study	Qualitative interview study	Not stated	Eight*	All experienced full MVC entrapment, requiring extrication by emergency responders	**Some features of DSM-5-TR diagnostic criteria for PTSD,** amnesia, avoidance,	Perceived threat to life, fear of fire, presence/absence of supportive human contact, memory disturbance, physical discomfort/pain, communication/involvement in extrication plans valued, debriefs/follow-up valued
Nutbeam, T [45]	2022	The development of evidence-based guidelines to inform the extrication of casualties trapped in motor vehicles following a collision	PhD Thesis	-	-	-	Refers to findings of the above qualitative interview study by Nutbeam et al	Refers to findings of the above qualitative interview study by Nutbeam et al
Doohan, I., Bjornstig, U., Ostlund, U. & Saveman, B.l [28]	2017	Exploring Injury Panorama, Consequences, and Recovery among Bus Crash Survivors: A Mixed-Methods Research Study**	Mixed-methods study	Bus	54 (out of total 56 survivors)	A minority experienced full entrapment; trapped/non-trapped ratio not declared	**31.5% of survivors at risk of PTSD as per Trauma Screening Questionnaire (TSQ)**, nightmares, flashbacks, feelings of depression	Clinically significant mental health outcomes, witnessing death, fear of fire, pain, presence or absence of supportive human contact, importance of clear communication

*14 eligible; 2 declined due perceived potential negative psychological consequences; 4 declined due to limited recall of their experiences

**Identified in secondary search but included in primary discussion due to high degree of relevance to primary question

Substantial heterogeneity exists across the included reports. Insights most specific to casualties’ experiences of MVC-entrapment stemmed from qualitative papers relying heavily on autobiographical accounts, therefore introducing potential confounds such as recall and publication biases. Where quantitative methods were employed, differences in study design, outcome measures, statistical analysis and absence of data regarding timing of psychological assessments further prohibit direct comparison. Despite such limitations, several overlapping themes were identified and are explored below.

### Summary of findings

Key findings have been drawn from a combination of case studies, qualitative literature and small-scale quantitative analyses. Despite a heterogeneity of sources, common psychological themes were noted across the included studies, with patterns suggesting that specific aspects of the entrapment experience may influence how psychological responses develop during and after the event.

### Casualties’ experience of MVC-entrapment

Across the included studies, casualties reported several common psychological experiences. Feelings of intense fear, uncertainty, and perceived threat to life were described consistently, particularly during periods in which casualties were unable to move or fully understand their situation [11, 28, 29]. Some reports highlighted a specific fear of vehicles catching fire and an inability to escape if this occurred [11, 28, 29]. Physical discomfort and pain were also salient themes, particularly when perceived as severe, prolonged, or poorly managed by rescue services [11, 28–31]. Many accounts highlighted feelings of helplessness and diminished autonomy, which appeared to negatively influence how individuals recalled and interpreted the event [11, 28, 29, 32]. In contrast, playing an active role in extrication or positive engagement with rescuers often resulted in less distress upon recall [11, 28–30, 32, 33]. Memory disturbance was another common experience, with some individuals reporting vivid, intrusive recollections and others describing partial or complete amnesia surrounding the event [11, 30, 34]. Other symptoms described following MVC-entrapment included nightmares, avoidance of reminders, heightened physiological arousal, and ongoing anxiety; in some cases meeting criteria for ASD or PTSD, as explored below.

### Clinically significant mental health outcomes following MVC-entrapment

Clinical diagnoses of ASD and PTSD were made in four case-studies of MVC-entrapment survivors [32, 34]. Further, 31.5% of survivors were considered at risk of PTSD (Trauma Screening Questionnaire (TSQ) score ≥ 6) following a bus crash with multiple trapped casualties (Trapped/Not-trapped ratio undisclosed) [28]. Finally, several DSM-5-TR diagnostic criteria for PTSD [35] were outlined by MVC-entrapment survivors who described features of ‘Recurrent distressing dreams’, ‘Efforts to avoid distressing memories, thoughts or feelings associated with the trauma’, ‘Avoidance of external reminders’ and ‘Inability to recall important aspects of the trauma’ [11, 30, 32, 34]. Finally, features of anxiety and depression were also described in a dual case study of two patients who had been trapped during an MVC [34].

### Bystander/Emergency medical services involvement

The value of human contact during entrapment and/or extrication was a recurrent theme throughout several papers, with survivors reporting the presence of either emergency services personnel or bystanders having improved their sense of safety and wellbeing [11, 28–30, 33].

Within this theme, several key observations stand out; the physical presence of another person prevented casualties feeling isolated, overlooked, or alone [11, 28–30, 33]. Simple verbal communication such as making distracting conversation and offering reassuring sentiments was also perceived positively [11, 28–30]. Furthermore, acts of compassion such as hand-holding or shielding from further harm were central, positive components of reported experiences during MVC-entrapment episodes [11, 28–30]. Casualties consistently showed positive regard for proactive, clear communication from rescue teams, as well as reporting increased distress where failures in such communication occurred [11, 28–30, 33]. Additionally, casualties valued planned follow up and post-accident debriefs when these occurred as it helped them process and understand their experience, as well serving to acknowledge the incident as an important life event [11].

### Contextual insights from secondary searches

In the absence of data specific to this review's primary question, secondary searches were used to explore more established fields of study where significant conceptual overlaps with the primary topic exist (MVCs in general and non-MVC forms of entrapment). PTSD, ASD, Depression and Anxiety were the most commonly reported mental health outcomes across both fields. The most robust prevalence findings for these outcomes are described below and offer a foundation from which to understand the potential psychological harms MVC-entrapment survivors are likely to sustain.

### Prevalence insights from secondary searches: MVCs in general

Our secondary searches revealed multiple robust sources exploring a range of adverse mental health outcomes following MVCs more generally. Multiple systematic literature reviews report significant heterogeneity of PTSD prevalence, ranging from 2.39–62% in adults [4, 13, 14] and 11–46% in children [4, 36]. Meta-analyses offer more focussed reporting of PTSD following MVCs in general, with pooled prevalence of 22.25% (95% CI 16.71%−28.33%) in adults [3] and 19.95% (95% CI 13.63% to 27.09%) in children [2].

Another meta-analysis described elevated rates of ASD where pooled prevalence was 21.51% (95% CI 11.82–33.08%) in adults, and 9.03% (95% CI: 2.90–17.89%), in children/adolescents following MVCs, generally [5].

Depression and anxiety following MVCs, generally were less robustly explored and no meta-analyses relating to these outcomes were found. One systematic literature review described the topic in depth but did not publish prevalence rates in the main text. Figures from the supplementary material showed prevalence findings between 8.5—32% for depression, and 7%—57% for anxiety [4].

### Prevalence insights from secondary searches: Non-MVC entrapment

Mental health outcomes following non-MVC events where entrapment was experienced have also been robustly explored, with most insights stemming from natural disaster settings and a large majority of this field addressing outcomes following earthquakes.

This field also displays significant heterogeneity in prevalence estimates for PTSD. One meta-analysis exploring risk factors for PTSD in survivors of earthquakes noted prevalence reports between 2.5%—60.0% in children and 4.1%—67.07% in adults. Significantly, they also concluded that being trapped during the earthquake was among the most significant risk factors for subsequent PTSD (pooled OR: Adults—1.81 (95% CI, 1.47–2.24); Children—1.94 (95% CI, 1.52–2.47)) [16].

No meta-analyses or literature reviews exploring ASD resulting from non-MVC entrapment exclusively were found, however one cohort study found 4.9% met DSM-IV diagnostic criteria for ASD with a further 39.3% meeting the threshold for ‘partial ASD’ (defined as showing at least one symptom on each DSM-IV criterion). Furthermore, 65.6% displayed ‘high levels of distress’ as measured by the 12 item General Health Questionnaire (GHQ-12). This study also found that having been trapped or injured under rubble was the strongest predictor of subsequent ASD [19].

A meta-analysis [18] of risk factors for depression in survivors of any form of natural disaster, also reported prevalence between 5.8%—54.0% in adults (12 earthquakes, six hurricanes/tornadoes, one tsunami and one flood) and 7.5%—44.8% in children/adolescents (nine earthquakes, one tornado and one tsunami). This meta-analysis was not limited to those who had been trapped, but did identify entrapment as a statistically significant risk factor for depression in children (pooled OR 1.73 (95% CI, 1.17–2.56).

No meta-analyses or literature reviews exploring anxiety following non-MVC forms of entrapment were found, however one cohort study reported 35% of those buried during an earthquake met diagnostic criteria for generalised anxiety disorder, three years later [22]. Another cohort study explored anxiety-related disorders, reporting 25% prevalence of agoraphobia and 20% prevalence of panic disorder in adult survivors who had been trapped under rubble following an earthquake [37]. Finally, a cohort study of adolescents reported 32.1% prevalence of anxiety after being involved in (but not necessarily trapped during) an earthquake [38].

## Discussion

Despite a small volume of heterogeneous evidence, this scoping review identifies consistent themes of fear, perceived threat to life, helplessness, and the value of supportive presence and clear communication, through the reported experiences of MVC-entrapment survivors. Diagnoses of ASD and PTSD were also highlighted where clinical scoring tools were employed.

### Interpretation of psychological mechanisms

The limited literature gives some insight into specific features of MVC-entrapment that may shape psychological responses during and after the incident. Fear of death, physical incapacity, restricted autonomy, and uncertainty appear particularly salient. Human contact is also important: calm, continuous communication and the presence of a supportive responder were experienced as protective, whereas isolation and poor situational understanding heightened distress. Although these observations cannot establish causation, they indicate that both environmental factors and interpersonal interactions during MVC-entrapment and subsequent rescue may influence survivors’ emotional responses.

### Inferences from wider literature on psychological outcomes following MVCs, generally

Exposure to MVCs in general is known to increase the risk of adverse mental health outcomes with fear, injury severity, perceived threat to life, and exposure to fatalities being some recognised risk factors [4, 13–15, 39–43]. Furthermore, research on physical outcomes from MVC-entrapment finds that trapped casualties are more likely to experience death and severe injury than those who can self-extricate [10]. In parallel, these findings suggest entrapment during MVCs may expose casualties to a greater number of established risk factors for adverse mental health compared to MVC without entrapment.

### Inferences from wider literature on psychological outcomes following non-MVC entrapment

Studies of entrapment in other contexts, such as natural disasters, also link injury, fear, perceived threat to life, witnessing injury or death, and prolonged immobility with a range of adverse mental health outcomes [16–18, 38, 44]. Although these settings differ from MVC entrapment in physical environment, duration, and rescue logistics, they share psychological mechanisms related to being unable to escape a potentially life-threatening situation. These conceptual parallels offer useful insights where MVC-specific evidence is scarce, although conclusions must remain cautious and context-specific.

### Clinical/Practical implications

Given the exploratory nature of the available evidence, the clinical implications identified in this review should be interpreted as hypothesis-generating rather than prescriptive. As summarised in Table 2, survivors consistently valued calm communication, reassurance, and a supportive human presence during entrapment and extrication.

**Table 2:** Clinical/Practical implications drawn from findings

Observation	Practical Implication
Survivors reported that **calm communication and reassurance** during extrication helped them cope	Emergency responders could provide continuous **calm, reassuring communication** to trapped individuals to help reduce acute distress
Having a **supportive person present** (responder or bystander) was valued by trapped casualties	Ensure a trained responder or **“extrication buddy”** stays with the casualty when possible, offering comfort (e.g. hand-holding, verbal support) throughout the rescue
**Psychological distress can persist** after hospital discharge (some trapped survivors developed PTSD/ASD, anxiety, etc.)	Provide **post-incident support or referrals** as needed: e.g. give patients information about signs of PTSD, arrange follow-up mental health screening, or signpost to counselling services
No evidence that changing **extrication techniques** (e.g. rapid vs. controlled extrication) alters psychological outcomes	**Focus on integrating psychological support** into standard extrication protocols rather than modifying physical rescue techniques. Training can highlight trauma-informed care (communication, empathy) during rescue

These elements can be integrated into routine practice without altering technical extrication strategies. Awareness that psychological distress may persist beyond hospital discharge highlights the potential value of signposting or follow-up where concerns arise. At present, the evidence does not support changes to extrication techniques themselves; rather, it supports embedding trauma-informed awareness alongside standard physical care.

### Strengths/Limitations

Strengths include an exhaustive primary search demonstrating a clear gap in the literature, and exploration of two conceptually related fields to contextualise this gap and identify recurring psychological mechanisms.

However, the subsequently small evidence base limits the predictive power of any hypotheses generated. The absence of studies directly comparing trapped and non-trapped MVC casualties limits the ability to determine entrapment-specific effects. Important contextual differences also exist between MVC-entrapment and other forms of entrapment. For example, earthquakes or building collapses often involve prolonged isolation within large, unstable structures requiring complex rescue, whereas MVCs involve smaller, more predictable structures with extrication typically completed within 30 min [45]. Socio-political factors further differentiate the two fields, with most existing MVC research coming from higher-income countries while, contrastingly, the majority of non-MVC entrapment research has been completed in lower-income countries. Hence, while non-MVC entrapment literature provides some foundational insights, important contextual differences limit transferability. Heterogeneity in study design, measurement tools, and timing of psychological assessments further constrains interpretation. These limitations are summarised in Table 3.

**Table 3:** Summary of key limitations of the scoping review and their impact

Limitation	Impact on Findings
**Limited evidence base** (only 7(+ 1) small studies; no quantitative synthesis)	Findings must be interpreted cautiously, and no causal inferences can be made from the available data
**No direct comparison of trapped vs. non-trapped survivors**	Cannot isolate entrapment-specific psychological effects (i.e. we don’t know if outcomes differ from non-trapped MVC survivors)
**Context differences from non-MVC entrapment** (e.g. earthquake vs. MVC)	Limits generalisability of insights from non-MVC settings; rescue contexts and survivor populations differ, so findings may not directly transfer
**Heterogeneity in study designs and measures**	Challenge to synthesise results; varying methodologies and assessment timing constrain how confidently we can interpret overall trends

### Recommendations for future research

Further research is required across several domains. In-depth analysis of risk factors established in conceptually related literature may extend the foundational insights of this review. Prospective cohort studies comparing psychological outcomes in trapped versus non-trapped MVC casualties are needed to clarify the specific impacts of MVC-entrapment on the types and severity of associated psychological harms. Examining associations between peri-traumatic variables—such as entrapment duration, extrication time, perceived threat to life, pain, and exposure to injury or death—would further refine our understanding in this area. Equally important is the exploration and evaluation of interventions tailored to trapped casualties, including the use of “extrication buddies” to mitigate peritraumatic distress, or research exploring the value of targeted post-traumatic interventions to address memory gaps and trauma-processing (e.g., structured debriefs, early contact with councillors, and trauma-informed therapies such as CBT and EMDR). Additional priorities include assessing the influence of pre-existing psychological conditions, the effects of different extrication strategies, and long-term recovery trajectories. Identifying protective factors that promote resilience may further inform intervention design.

## Conclusions

Direct evidence regarding psychological sequelae of entrapment following MVC is severely limited. The available evidence suggests that MVC-entrapment may be associated with significant psychological harms and may place survivors at increased risk of clinically significant sequelae such as ASD and PTSD. Simple, yet effective interventions such as facilitated self-extrication (where practicable) or the deployment of extrication buddies may mitigate the impact of some harmful elements of the MVC-entrapment experience, thereby reducing the burden of psychological harms sustained. This review highlights the urgent need for improved understanding across this topic and provides a foundation for further research which should focus on establishing causality and developing evidence-based approaches to psychological care during and after MVC-entrapment.

## Supplementary Information

Supplementary Material 1.

## Data Availability

All data generated or analysed during this study are included in this published article and its supplementary materials.

## Abbreviations

ASD: Acute stress disorder
CBT: Cognitive behavioural therapy
CI: Confidence interval
DSM-*: Diagnostic and statistical manual of mental disorders-*(edition)
EMDR: Eye movement desensitisation and reprocessing
GHQ-12: 12-Item General health questionnaire
MVC: Motor vehicle collision
NHS: National health service (UK)
OR: Odds ratio
PRISMA: Preferred Reporting Items for Systematic Reviews and Meta-Analyses
PRISMA-ScR: PRISMA extension for scoping reviews
PTSD: Post traumatic stress disorder
TSQ: Trauma screening questionnaire
