# Supplementary material for: Trapped and traumatised: a scoping review of the psychological sequelae of entrapment following motor vehicle collision
Source: Scand J Trauma Resusc Emerg Med. 2026 Jan 29;34:49. doi: 10.1186/s13049-026-01558-9 (PMC12947406; doi:10.1186/s13049-026-01558-9)
Supplement: Supplementary file 1 — Supplementary Material 1. [file 13049_2026_1558_MOESM1_ESM.docx]

**Supplementary material**

**List of Appendices**

**Appendix 1** Pages 2-3

Search strategy and search terms, primary question: Does entrapment (and/or extrication by emergency services) following motor vehicle collision (MVC) lead to worse psychological/mental health outcomes compared to motor vehicle accidents/collisions without entrapment?

**Appendix 2** Pages 4-6

Search strategy, search terms and Figure 2, PRISMA diagram for secondary question (a): What are the mental health outcomes associated with involvement in motor vehicle collisions in general?

**Appendix 3** Pages 7-9

Search strategy, search terms and Figure 3, PRISMA diagram for secondary question (b): What are the mental health outcomes associated with non-MVC entrapment?

**Appendix 4** Pages 10-14

Summary tables for papers reporting prevalence of mental health conditions following MVCs in general (i.e. without specific mention of entrapment).

**Appendix 5** Pages 15-20

Summary tables for papers reporting prevalence of mental health conditions following non-MVC events where entrapment was common or likely.

**Appendix 6** Pages 21-22

PRISMA-ScR reporting checklist

**Appendix 1: Search strategy and search terms for primary question:**

**Does entrapment (and/or extrication by emergency services) following motor vehicle collision (MVC) lead to worse psychological/mental health outcomes compared to motor vehicle accidents/collisions without entrapment?**

Appendix 1.1 - Search terms used in NHS Knowledge and Library Hub^^[[1]](#footnote-1)^^

| S1 | TI ( (road or traffic or motor or car* or vehicle*) N2 (accident* or collision* or crash*) ) OR AB ( (road or traffic or motor or car* or vehicle*) N2 (accident* or collision* or crash*) ) OR SU ( (road or traffic or motor or car* or vehicle*) N2 (accident* or collision* or crash*) ) |
| --- | --- |
| S2 | TI ( entrapment or trapped or rescue* or extricat* ) OR AB ( entrapment or trapped or rescue* or extricat* ) OR SU ( entrapment or trapped or rescue* or extricat* ) |
| S3 | TI ( "mental health" or psychological or psychiatric or "post-traumatic stress" or "posttraumatic stress" or pts or ptsd or depression or depressed or anxiety or panic or phobia* or "quality of life" or wellbeing or "mental state" or "trauma-induced stress" ) OR AB ( "mental health" or psychological or psychiatric or "post-traumatic stress" or "posttraumatic stress" or pts or ptsd or depression or depressed or anxiety or panic or phobia* or "quality of life" or wellbeing or "mental state" or "trauma-induced stress" ) OR SU ( "mental health" or psychological or psychiatric or "post-traumatic stress" or "posttraumatic stress" or pts or ptsd or depression or depressed or anxiety or panic or phobia* or "quality of life" or wellbeing or "mental state" or "trauma-induced stress" ) |
| S4 | S1 AND S2 AND S3 |

Appendix 1.2 - Search terms used in Proquest (PsycArticles, PsycInfo, British Nursing Index, Health Research Premium Collection, PTSDPubs)

|  | |
| --- | --- |
| S1 | tiabsu((road OR traffic OR motor OR car* OR vehicle*) NEAR/2 (accident* OR collision* OR crash*)) AND tiabsu(entrapment OR trapped OR rescue* OR extricat*) AND tiabsu("mental health" OR psychological OR psychiatric OR "post-traumatic stress" OR "posttraumatic stress" OR pts OR ptsd OR depression OR depressed OR anxiety OR panic OR phobia* OR "quality of life" OR wellbeing OR "mental state" OR "trauma-induced stress") |

Appendix 1.3 - Search terms used in Embase and Medline via Ovid

|  | |
| --- | --- |
| 1 | exp Accidents, Traffic/ |
| 2 | ((road or traffic or motor or car* or vehicle*) adj3 (accident* or collision* or crash*)).mp. [mp=ti, ab, hw, tn, ot, dm, mf, dv, kf, fx, dq, bt, nm, ox, px, rx, ui, sy, ux, mx] |
| 3 | 1 or 2 |
| 4 | (entrapment or trapped or extricat*).mp. [mp=ti, ab, hw, tn, ot, dm, mf, dv, kf, fx, dq, bt, nm, ox, px, rx, ui, sy, ux, mx] |
| 5 | exp Stress Disorders, Post-Traumatic/ or exp Mental Disorders/ or exp Mental Health/ |
| 6 | (“mental health” or psychological or psychiatric or “post-traumatic stress” or “posttraumatic stress” or pts or ptsd or depression or depressed or anxiety or panic or phobia* or “quality of life” or wellbeing or “mental state” or trauma-induced stress”).mp. [mp=ti, ab, hw, tn, ot, dm, mf, dv, kf, fx, dq, bt, nm, ox, px, rx, ui, sy, ux, mx] |
| 7 | 5 or 6 |
| 8 | 3 and 4 and 7 |

**Appendix 2 – Search strategy, search terms and PRISMA diagram, secondary question (a):**

**What are the mental health outcomes associated with involvement in motor vehicle collisions in general?**

Appendix 2.1 - Search terms used in NHS Knowledge and Library Hub^^[[2]](#footnote-2)^^

|  | |
| --- | --- |
| S1 | TI ((road or traffic or motor or car* or vehicle*) N2 (accident* or collision* or crash*)) OR AB ( (road or traffic or motor or car* or vehicle*) N2 (accident* or collision* or crash*) ) OR SU ( (road or traffic or motor or car* or vehicle*) N2 (accident* or collision* or crash*) ) |
| S2 | TI ( "mental health" or psychological or psychiatric or "post-traumatic stress" or "posttraumatic stress" or pts or ptsd or depression or depressed or anxiety or panic or phobia* or "quality of life" or wellbeing or "mental state" or "trauma-induced stress" ) OR AB ( "mental health" or psychological or psychiatric or "post-traumatic stress" or "posttraumatic stress" or pts or ptsd or depression or depressed or anxiety or panic or phobia* or "quality of life" or wellbeing or "mental state" or "trauma-induced stress" ) OR SU ( "mental health" or psychological or psychiatric or "post-traumatic stress" or "posttraumatic stress" or pts or ptsd or depression or depressed or anxiety or panic or phobia* or "quality of life" or wellbeing or "mental state" or "trauma-induced stress" ) |
| S3 | S1 AND S2 |

Appendix 2.2 - Search terms used in Proquest (PsycArticles, PsycInfo, British Nursing Index, Health Research Premium Collection, PTSDPubs)

|  | |
| --- | --- |
| S1 | (tiabsu((road OR traffic OR motor OR car* OR vehicle*) NEAR/2 (accident*  OR collision* OR crash*)) AND tiabsu("mental health" OR psychological OR  psychiatric OR "post-traumatic stress" OR "posttraumatic stress" OR pts  OR ptsd OR depression OR depressed OR anxiety OR panic OR phobia*  OR "quality of life" OR wellbeing OR "mental state" OR "trauma-induced  stress")) AND (subt.exact("posttraumatic stress disorder" OR "stress  disorders, post-traumatic" OR "mental health" OR "ptsd (dsm-iv)" OR  "mental disorders" OR "post traumatic stress disorder" OR "ptsd" OR  "anxiety" OR "depressive disorders" OR "emotional trauma" OR "anxiety  disorders" OR "stress" OR "adaptation, psychological" OR "acute stress  disorder" OR "suicide" OR "mental depression" OR "psychiatric status  rating scales" OR "major depression" OR "stress, psychological" OR  "emotions" OR "ptsd (dsm-iii-r)") AND pd(20140112-20240112)) AND  (subt.exact(("motor traffic accidents" OR "traffic accidents & safety" OR  "accidents, traffic" OR "accidents" OR "motor vehicles" OR "traffic" OR  "vehicles" OR "traffic accidents" OR "automobile driving" OR "drivers") AND  "mental health") AND pd(20040101-20231231) AND PEER(yes)) |
|  |  |

Appendix 2.3 - Search terms used in Embase & Medline via Ovid

| Search 2 - What are the mental health outcomes associated with involvement in a motor vehicle accident/collision? | |
| --- | --- |
| 1 | exp Accidents, Traffic / or exp traffic accident/ |
| 2 | ((road or traffic or motor or car* or vehicle*) adj3 (accident* or collision* or crash)).tw. |
| 3 | 1 or 2 |
| 4 | exp Stress Disorders, Post-Traumatic/ or exp Mental Disorders/ or exp Mental Health/ |
| 5 | (“mental health” or psychological or psychiatric or “post-traumatic stress” or “posttraumatic stress” or pts or ptsd or depression or depressed or anxiety or panic or phobia* or “quality of life” or wellbeing or “mental state” or “trauma-induced stress”).tw. |
| 6 | 4 or 5 |
| 7 | 3 and 6 |

*Figure 2: PRISMA diagram[27] outlining study selection process* for secondary question (a): What are the mental health outcomes associated with involvement in motor vehicle collisions in general?


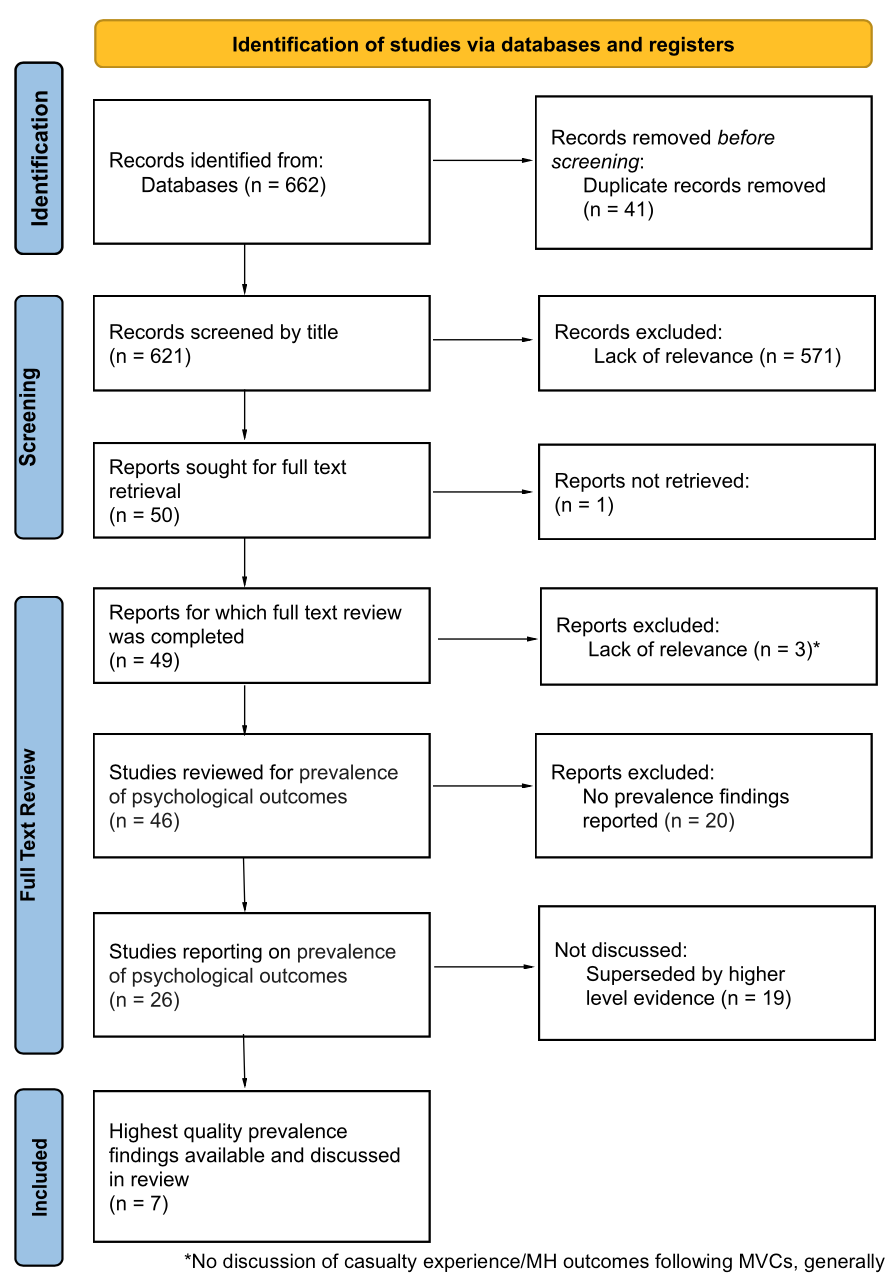


**Appendix 3 - Search strategy, search terms and PRISMA diagram, secondary question (b):**

**What are the mental health outcomes associated with non-MVC entrapment?**

Appendix 3.1 - Search terms used in NHS Knowledge & Library Hub^^[[3]](#footnote-3)^^

|  | |
| --- | --- |
| S1 | TI ( Disaster* OR Earthquake OR (Building* N2 collaps*) OR cave* OR caving OR potholing OR Flood* OR Hurricane* OR Rockfall* OR Mining* OR mines OR Avalanche* OR Landslide*) ) OR AB ( Disaster* OR Earthquake OR (Building* N2 collaps*) OR cave* OR caving OR potholing OR Flood* OR Hurricane* OR Rockfall* OR Mining* OR mines OR Avalanche* OR Landslide*) ) OR SU ( Disaster* OR Earthquake OR (Building* N2 collaps*) OR cave* OR caving OR potholing OR Flood* OR Hurricane* OR Rockfall* OR Mining* OR mines OR Avalanche* OR Landslide*) ) |
| S2 | TI ( "mental health" OR psychological OR psychiatric OR "post-traumatic stress" OR "posttraumatic stress" OR pts OR ptsd OR depression OR depressed OR anxiety OR panic OR phobia* OR "quality of life" OR wellbeing OR "mental state" OR "trauma-induced stress" ) OR AB ( "mental health" OR psychological OR psychiatric OR "post-traumatic stress" OR "posttraumatic stress" OR pts OR ptsd OR depression OR depressed OR anxiety OR panic OR phobia* OR "quality of life" OR wellbeing OR "mental state" OR "trauma-induced stress" ) OR SU ( "mental health" OR psychological OR psychiatric OR "post-traumatic stress" OR "posttraumatic stress" OR pts OR ptsd OR depression OR depressed OR anxiety OR panic OR phobia* OR "quality of life" OR wellbeing OR "mental state" OR "trauma-induced stress" ) |
| S3 | TI ( entrapment OR trapped OR extricat*) ) OR AB ( entrapment OR trapped OR extricat*) ) OR SU ( entrapment OR trapped OR extricat*) ) |
| S4 | S1 AND S2 AND S3 |

Appendix 3.2 - Search terms used in Proquest (PsycArticles, PsycInfo, British Nursing Index, Health Research Premium Collection, PTSDPubs)

|  | |
| --- | --- |
| S1 | tiabsu(Disaster* OR Earthquake OR (Building* NEAR/2 collaps*) OR cave*  OR caving OR potholing OR Flood* OR Hurricane* OR Rockfall* OR  Mining* OR mines OR Avalanche* OR Landslide*) AND tiabsu("mental  health" OR psychological OR psychiatric OR "post-traumatic stress" OR  "posttraumatic stress" OR pts OR ptsd OR depression OR depressed OR  anxiety OR panic OR phobia* OR "quality of life" OR wellbeing OR "mental  state" OR "trauma-induced stress") AND tiabsu(entrapment OR trapped OR  extricat*) AND pd(20040101-20240112) |

Appendix 3.3 - Search terms used in Embase and Medline via Ovid

|  | |
| --- | --- |
| 1 | (entrapment or trapped or extricat*).mp. [mp=ti, ab, hw, tn, ot, dm, mf, dv, kf, fx, dq, bt, nm, ox, px, rx, ui, sy, ux, mx] |
| 2 | exp Stress Disorders, Post-Traumatic/ or exp Mental Disorders/ or exp Mental Health/ |
| 3 | (“mental health” or psychological or psychiatric or “post-traumatic stress” or “posttraumatic stress” or pts or ptsd or depression or depressed or anxiety or panic or phobia* or “quality of life” or wellbeing or “mental state” or “trauma-induced stress”).mp. [mp=ti, ab, hw, tn, ot, dm, mf, dv, kf, fx, dq, bt, nm, ox, px, rx, ui, sy, ux, mx] |
| 4 | 2 or 3 |
| 5 | (Disaster* or Earthquake or (Building* adj3 collaps*) or cave* or caving or potholing or Flood* or Hurricane or Rockfall* or Mining* or mines or Avalanche* or Landslide*).mp. [mp=ti, ab, hw, tn, ot, dm, mf, dv, kf, fx, dq, bt, nm, ox, px, rx, ui, sy, ux, mx] |
| 6 | 1 and 4 and 5 |

*Figure 3: PRISMA diagram[27] outlining study selection process* for secondary question (b):

What are the mental health outcomes associated non-MVC entrapment?


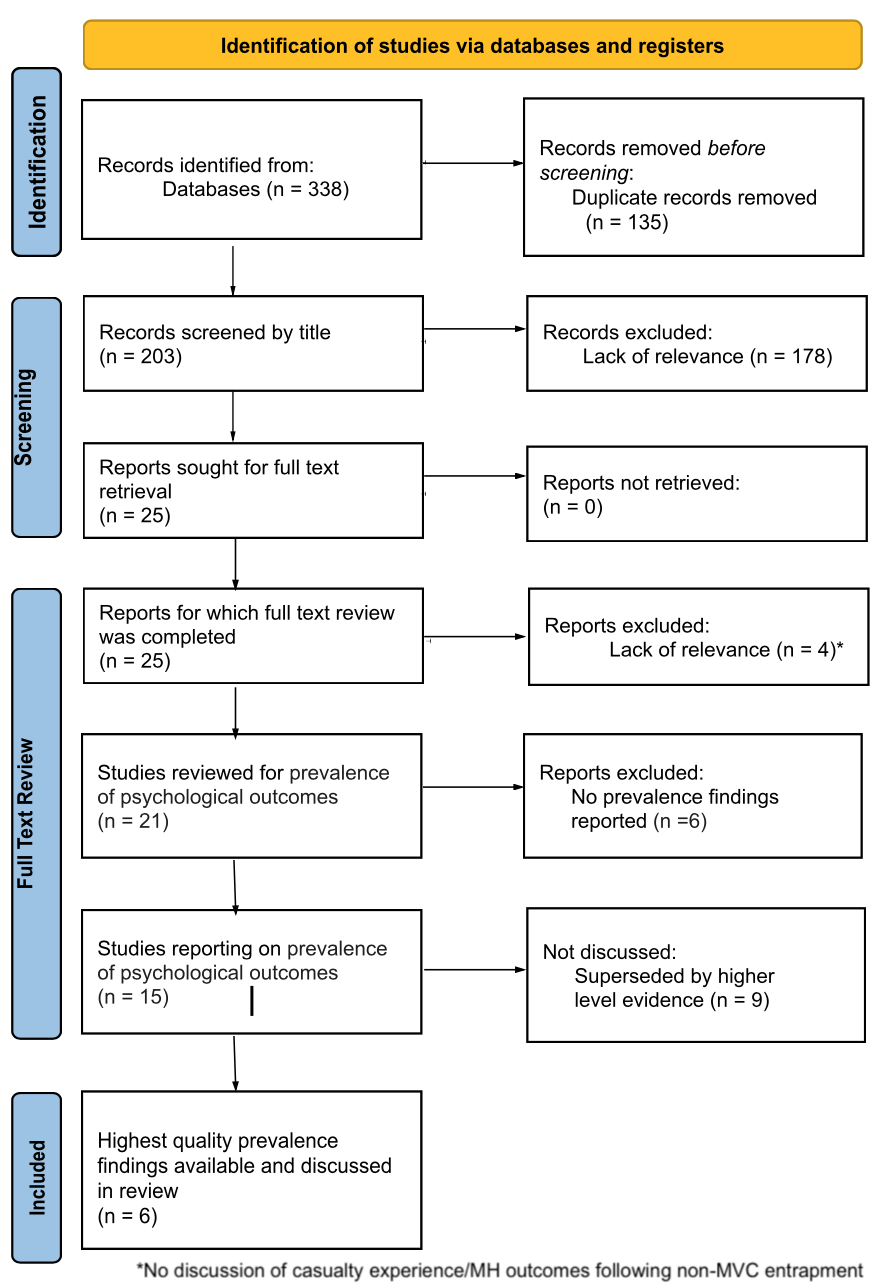


| ***Appendix 4 - Table 4.1. Summary of papers reporting PTSD prevalence following MVC*** | | | | | |  |
| --- | --- | --- | --- | --- | --- | --- |
| **Author** | **Year of Publication** | **Title** | **Article Type** | **Prevalence finding** | **Timescale from MVC, notes on findings** | |
| **ADULTS** | | | | | |  |
| Kuhn, E., Blanchard, E. B., Fuse, T., Hickling, E. J. & Broderick, J. | 2006 | Heart rate of motor vehicle accident survivors in the emergency department, peritraumatic psychological reactions, ASD, and PTSD severity: A 6‐month prospective study. | Single study | 8.0%-14.0% | 14.0% at 1 month, 8.0% at 3 months, 8.0% at 6 months | |
| Smith, B., Mackenzie-Ross, S. & Scragg, P. | 2007 | Prevalence of poor psychological morbidity following a minor road traffic accident (RTA): The clinical implications of a prospective longitudinal study. | Single study | 12.8%-14.0% | 14.0% at 1 month; 12.8% at 4 months | |
| Ehring, T., Ehlers, A. & Glucksman, E. | 2008 | Do Cognitive Models Help in Predicting the Severity of Posttraumatic Stress Disorder, Phobia, and Depression After Motor Vehicle Accidents? A Prospective Longitudinal Study. | Single study | 12.1%-22.4% | 22.4 % at 2 weeks, 12.1% at 6 months | |
| Ryb, G. E., Dischinger, P. C., Read, K. M. & Kufera, J. A. | 2009 | PTSD after severe vehicular crashes. | Single study | 24.3%-27.5% | 27.5% at 6 months; 24.3% at 12 months | |
| Chossegros, L. et al. | 2011 | Predictive factors of chronic post-traumatic stress disorder 6 months after a road traffic accident. | Single study | 18.5% | 6 months | |
| Coronas, R. et al. | 2011 | Heart rate measured in the acute aftermath of trauma can predict post-traumatic stress disorder: A prospective study in motor vehicle accident survivors. | Single study | 32.8%-45.4% | 45.4% at 1 month; 32.8% at 4 months | |
| Heron-Delaney, M., Kenardy, J., Charlton, E. & Matsuoka, Y. | 2013 | A systematic review of predictors of posttraumatic stress disorder (PTSD) for adult road traffic crash survivors. | SLR | 6%-45% | 1 month to 5 years | |
| Hruska, B., Irish, L. A., Pacella, M. L., Sledjeski, E. M. & Delahanty, D. L. | 2014 | PTSD symptom severity and psychiatric comorbidity in recent motor vehicle accident victims: A latent class analysis. | Single study | 10.0% | 6 weeks | |
| Hiller, R. M. et al. | 2016 | Predictors of Posttraumatic Stress Symptom Trajectories in Parents of Children Exposed to Motor Vehicle Collisions. | Single study | 2%-58% | PTSD symptoms in parents of children involved in MVC; variation by severity and time: <4 weeks; 47% mild, 24% moderate, 15% moderate-severe, 3% severe; 6 months; 54% mild, 13% moderate, 9% mod-severe, 2% severe: 3 years; 58% mild, 6% moderate, 2% mod-severe, 0% severe. | |
| Doohan, I., Björnstig, U., Östlund, U. & Saveman, B.-I. | 2017 | Exploring Injury Panorama, Consequences, and Recovery among Bus Crash Survivors: A Mixed-Methods Research Study. | Single study | 31% (“high risk” for developing PTSD; | 1-3 months | |
| Lin, W., Gong, L., Xia, M. & Dai, W. | 2018 | Prevalence of posttraumatic stress disorder among road traffic accident survivors. | Meta-Analysis | 22.3% | 1 month to 37 years | |
| Kovacevic, J. et al. | 2020 | Predictors of Mental Health Outcomes in Road Traffic Accident Survivors. | Single study | 32.3% | 1 month | |
| Montes, S. A. & Ledesma, R. D. | 2021 | Post-traumatic stress disorder after road traffic accidents: a systematic review. | SLR | 2.39%-62% (95% CI 16.71%-28.33%) | abstract only, main paper in Spanish so unable to determine further details | |
| Soori, H., Yousefinezhadi, T. & Yoousefinezhadi, T. | 2021 | Epidemiologic Study of Post-Traumatic Stress Disorder (PTSD) among Traffic Accident Victims in Tehran. Iran. | Single study | 40.6% | 2 months | |
| Ziobrowski, H. N. et al. | 2021 | Development and Validation of a Model to Predict Posttraumatic Stress Disorder and Major Depression After a Motor Vehicle Collision. | Single study | 25.1% | 3 months | |
| Marasini, G., Caleffi, F., Machado, L. M. & Pereira, B. M. | 2022 | Psychological consequences of motor vehicle accidents: A systematic review. | SLR | 7.9%-36% | 1-24 months | |
| **CHILDREN/ADOLESCENTS** | | | | | |  |
| Stallard, P., Salter, E. & Velleman, R. | 2004 | Posttraumatic stress disorder following road traffic accidents: A second prospective study. | Single Study | 29.1% | 4 weeks | |
| Pervanidou, P. et al. | 2007 | The Natural History of Neuroendocrine Changes in Pediatric Posttraumatic Stress Disorder (PTSD) After Motor Vehicle Accidents: Progressive Divergence of Noradrenaline and Cortisol Concentrations Over Time. | Single Study | 15%-38.3% | 38.3% at 1 month, 15% at 6 months | |
| Kolaitis, G. et al. | 2011 | Predicting pediatric posttraumatic stress disorder after road traffic accidents: The role of parental psychopathology. | Single study | 18.8%-40.4% | 40.4% at 1 month; 18.8% at 6 months | |
| Mehta, S. & Ameratunga, S. N. | 2012 | Prevalence of post‐traumatic stress disorder among children and adolescents who survive road traffic crashes: A systematic review of the international literature. | SLR | 12%-46% | 12-46% at <4 months post MVC, reducing to 13%-25% between 4-12 months post MVC; some variability noted to be based on parent vs child reporting of child symptoms | |
| Tierens, M. et al. | 2012 | Differences in posttraumatic stress reactions between witnesses and direct victims of motor vehicle accidents. | Single Study | 11.19%-14.16% | 1 month - >1 year post MVC Witnesses 11.19%, victims 14.16% | |
| Williams, J. L., Rheingold, A. A., Knowlton, A. W., Saunders, B. E. & Kilpatrick, D. G. | 2015 | Associations Between Motor Vehicle Crashes and Mental Health Problems: Data From the National Survey of Adolescents‐Replication. | Single Study | 7.4% | Lifetime involvement in MVC, average age at MVC of 10 years | |
| Dai, W. et al. | 2018 | Prevalence of Posttraumatic Stress Disorder among Children and Adolescents following Road Traffic Accidents: A Meta-Analysis. | Meta-Analysis | 20.0% | 1-18 months | |
| Yoshino, M. et al. | 2022 | Post-Traumatic Stress Disorder among Children Involved in Traffic Accidents and Their Parents in Japan. | Single Study | 10.1% | mean post-MVC period 33 months, SD 17.40 months | |
| Marasini, G., Caleffi, F., Machado, L. M. & Pereira, B. M. | 2022 | Psychological consequences of motor vehicle accidents: A systematic review. | SLR | 11%-37.1% | ‘baseline’ and 8 months | |

| ***Appendix 4 - Table 4.2. Summary of papers reporting ASD prevalence following MVC*** | | | | | |
| --- | --- | --- | --- | --- | --- |
| **Author** | **Year of Publication** | **Title** | **Article Type** | **Prevalence finding** | **Timescale from MVC** |
| **ADULTS** | | | | | |
| Kuhn, E., Blanchard, E. B., Fuse, T., Hickling, E. J. & Broderick, J. | 2006 | Heart rate of motor vehicle accident survivors in the emergency department, peritraumatic psychological reactions, ASD, and PTSD severity: A 6‐month prospective study. | Single Study | 10% (a further 28% showed sub-diagnostic levels - all but dissociative symptoms) | 2 Weeks |
| Dai, W. et al. | 2018 | Prevalence of acute stress disorder among road traffic accident survivors: a meta-analysis. | Meta-Analysis | 21.51% (95% CI: 11.82– 33.08%) | 2 days to 1 month |
| Soori, H., Yousefinezhadi, T. & Yoousefinezhadi, T. | 2021 | Epidemiologic Study of Post-Traumatic Stress Disorder (PTSD) among Traffic Accident Victims in Tehran. | Single Study | 49% | 1 Week |
| **CHILDREN/ADOLESCENTS** | | | | | |
| Dai, W. et al. | 2018 | Prevalence of acute stress disorder among road traffic accident survivors: a meta-analysis. | Meta-Analysis | 9.03% (95% CI: 2.90–17.89%) | 2 day to 1 month |
| Marasini, G., Caleffi, F., Machado, L. M. & Pereira, B. M. | 2022 | Psychological consequences of motor vehicle accidents: A systematic review. | SLR | 15% | Timescale not stated (by definition would be between 1 day-1 month) |

| ***Appendix 4 - Table 4.3. Summary of papers reporting Depression prevalence following MVC*** | | | | | |
| --- | --- | --- | --- | --- | --- |
| **Author** | **Year of Publication** | **Title** | **Article Type** | **Prevalence finding** | **Timescale from MVC** |
| **ADULTS** | | | | | |
| Smith, B., Mackenzie-Ross, S. & Scragg, P. | 2007 | Prevalence of poor psychological morbidity following a minor road traffic accident (RTA): The clinical implications of a prospective longitudinal study. | Single Study | 10.0%-10.3% | 10.0% at 1 month, 10.3% at 4 months |
| Ehring, T., Ehlers, A. & Glucksman, E. | 2008 | Do Cognitive Models Help in Predicting the Severity of Posttraumatic Stress Disorder, Phobia, and Depression After Motor Vehicle Accidents? A Prospective Longitudinal Study. | Single Study | 7-8%-9.6% | 9.6% at 2 weeks, 7.8% at 6 months |
| Hruska, B., Irish, L. A., Pacella, M. L., Sledjeski, E. M. & Delahanty, D. L. | 2014 | PTSD symptom severity and psychiatric comorbidity in recent motor vehicle accident victims: A latent class analysis. | Single Study | 12.60% | 6 weeks |
| Kovacevic, J. et al. | 2020 | Predictors of Mental Health Outcomes in Road Traffic Accident Survivors. | Single Study | 17.40% | 1 month |
| Ziobrowski, H. N. et al. | 2021 | Development and Validation of a Model to Predict Posttraumatic Stress Disorder and Major Depression After a Motor Vehicle Collision. | Single Study | 11.50% | 3 months |
| Joormann J, McLean SA, Beaudoin FL, et al. | 2022 | Socio-demographic and trauma-related predictors of depression within eight weeks of motor vehicle collision in the AURORA study. | Single Study | 27.8% | 8 weeks |
| Marasini, G., Caleffi, F., Machado, L. M. & Pereira, B. M. | 2022 | Psychological consequences of motor vehicle accidents: A systematic review. | SLR | 8.5%-32% (values manually extracted from supplemental materials) | 1-18 months |
| **CHILDREN/ADOLESCENTS** | | | | | |
| Stallard, P., Salter, E. & Velleman, R. | 2004 | Posttraumatic stress disorder following road traffic accidents: A second prospective study. Eur. Child Adolesc. | Single Study | 17.70% | 4 weeks |
| Williams, J. L., Rheingold, A. A., Knowlton, A. W., Saunders, B. E. & Kilpatrick, D. G. | 2015 | Associations Between Motor Vehicle Crashes and Mental Health Problems: Data From the National Survey of Adolescents‐Replication. | Single Study | 11.20% | Lifetime involvement in MVC, average age at MVC of 10 years |
| Marasini, G., Caleffi, F., Machado, L. M. & Pereira, B. M. | 2022 | Psychological consequences of motor vehicle accidents: A systematic review. | SLR | 13.8%-15.5% (SLR, reporting one study only (Stallard et al 2001a), | 15.5% at 6 weeks, 13.8% at 8 months |

| ***Appendix 4 - Table 4.4. Summary of papers reporting Anxiety prevalence following MVC*** | | | | | |
| --- | --- | --- | --- | --- | --- |
| **Author** | **Year of Publication** | **Title** | **Article Type** | **Prevalence finding** | **Timescale from MVC** |
| **ADULTS** | | | | | |
| Smith, B., Mackenzie-Ross, S. & Scragg, P. | 2007 | Prevalence of poor psychological morbidity following a minor road traffic accident (RTA): The clinical implications of a prospective longitudinal study. | Single Study | 17.9%-24% moderate-high levels of anxiety, | 24% at 1 month, 17.9% at 4 months, with a further 36%-38% reporting ‘symptoms of anxiety’ while driving, reducing with time from baseline to 6 months |
| Kovacevic, J. et al. | 2020 | Predictors of Mental Health Outcomes in Road Traffic Accident Survivors. | Single Study | 5.80% | 1 month |
| Marasini, G., Caleffi, F., Machado, L. M. & Pereira, B. M. | 2022 | Psychological consequences of motor vehicle accidents: A systematic review. | SLR | 7%-57% SLR (figures from supplementary material) | Baseline' to 5 years |
| **CHILDREN/ADOLESCENTS** | | | | | |
| Stallard, P., Salter, E. & Velleman, R. | 2004 | Posttraumatic stress disorder following road traffic accidents: A second prospective study. | Single Study | 20.30% | 4 weeks |
| Marasini, G., Caleffi, F., Machado, L. M. & Pereira, B. M. | 2022 | Psychological consequences of motor vehicle accidents: A systematic review. | SLR | 11.1%-14.7% (SLR, reporting one study only (Stallard et al 2001a) | 14.7% at 6 weeks, 11.1% at 8 months |

| ***Appendix 5 - Table 5.1. Summary of papers reporting PTSD prevalence following non-MVC events where entrapment was common or likely*** | | | | | | | |
| --- | --- | --- | --- | --- | --- | --- | --- |
| **Author** | **Year of Publication** | **Title** | **Article Type** | **Entrapment specific?** | **Event** | **Prevalence finding** | **Timescale from event** |
| **ADULTS** | | | | | | | |
| Çorapçloğlu, A., Tural, Ü., Yargiç, I. & Kocabaşoğlu, N. | 2004 | Subthreshold post traumatic stress disorder in the survivors of Marmara earthquake. | Single study | No but entrapment identified as significant risk factor | Earthquake | 13.8% ‘full PTSD’-28% ‘sub-theshold’ PTSD’ | Timescale not stated |
| Wang, H.-H. et al. | 2010 | Psychopathological, biological, and neuroimaging characterization of posttraumatic stress disorder in survivors of a severe coalmining disaster in China. | Single study | Yes | Mining disaster | 35.4% at 3 months, 31.6% at 6 months | 3 months-6 months |
| Aziz, S. & Aslam, N. | 2012 | Psychiatric Morbidity and Work and Social Adjustment Among Earthquake Survivors Extricated from under the Rubble. | Single study | Yes | Earthquake | 32.5% | 6 years |
| Qi, S. et al. | 2013 | Cortical inhibition deficits in recent onset PTSD after a single prolonged trauma exposure | Single study | Yes | Mining disaster | 35.42% | 6 months |
| Cerdá, M. et al. | 2013 | PSYCHOPATHOLOGY IN THE AFTERMATH OF THE HAITI EARTHQUAKE: A POPULATION‐BASED STUDY OF POSTTRAUMATIC STRESS DISORDER AND MAJOR DEPRESSION. | Single study | No but entrapment identified as significant risk factor | Earthquake | 24.6%, 2-4 months after earthquake | 2 months-4 months |
| Tang B, Deng Q, Glik D, et al. | 2017 | A Meta-Analysis of Risk Factors for Post-Traumatic Stress Disorder (PTSD) in Adults and Children after Earthquakes. | Meta-Analysis | No but entrapment identified as significant risk factor | Earthquake | 4.1%-67.07% (MA examining risk factors, so pooled prevalence not generated) | 1 month - 60 months |
| Abolhadi, E., Divsalar, P., Mosleh-Shirazi, M. A. & Dehesh, T. | 2022 | Latent classes of posttraumatic stress disorder among survivors of the Bam Earthquake after 17 years. | Single study | No but entrapment identified as significant risk factor | Earthquake | 19.9% | 17 years |
| Petrucci, E. et al. | 2023 | Health Status Perception and Psychological Sequelae in Buried Victims: An Observational Study on Survivors of the Earthquake in Amatrice (Italy), Three Years Later. | Single study | Yes | Earthquake | 57% | 3 years |
| **CHILDREN/ADOLESCENTS** | | | | | | | |
| Eksi, A. & Braun, K. L. | 2009 | Over-time changes in PTSD and depression among children surviving the 1999 Istanbul earthquake. | Single Study | No but having being in serious personal danger (“e.g. trapped in a building or under rubble” was the only significant predictor of persistent PTSD | Earthquake | 60% at baseline, 18% of those still met diagnostic threshold for PTSD at 18-20 months | Baseline - 18 months-20 months |
| Tang B, Deng Q, Glik D, et al. | 2017 | A Meta-Analysis of Risk Factors for Post-Traumatic Stress Disorder (PTSD) in Adults and Children after Earthquakes. | Meta-Analysis | No but entrapment identified as significant risk factor | Earthquake | 2.5%-60.0% (MA examining risk factors, so pooled prevalence not generated) | 1 month - 36 months |
| Sharma, A. & Kar, N. | 2019 | Posttraumatic Stress, Depression, and Coping Following the 2015 Nepal Earthquake: A Study on Adolescents. | Sinlge Study | No but entrapment identified as significant risk factor | Earthquake | 43.4% | 1 year |
| Marthoenis, M., Ilyas, A., Sofyan, H. & Schouler-Ocak, M. | 2019 | Prevalence, comorbidity and predictors of post-traumatic stress disorder, depression, and anxiety in adolescents following an earthquake. | Single Study | No but witnessing someone being trapped or injured was identified as risk factor | Earthquake | 58.3% | 6 months |
| Qi, J., Yang, X., Tan, R., Wu, X. & Zhou, X. | 2020 | Prevalence and predictors of posttraumatic stress disorder and depression among adolescents over 1 year after the Jiuzhaigou earthquake. | Single Study | No but entrapment identified as significant risk factor, as was having having friends/family who were trapped | Earthquake | 46.3% | 1 year |
| Wahab, S. et al. | 2021 | Post-traumatic Stress Symptoms in Adolescents Exposed to the Earthquake in Lombok, Indonesia: Prevalence and Association With Maladaptive Trauma-Related Cognition and Resilience. | Single Study | No but entrapment identified as significant risk factor | Earthquake | 69.9% “PTS symptoms” | 5 months-12 months |

| ***Appendix 5 - Table 5.2 - Summary of papers reporting ASD prevalence following non-MVC events where entrapment was common or likely*** | | | | | | | |
| --- | --- | --- | --- | --- | --- | --- | --- |
| **Author** | **Year of Publication** | **Title** | **Article Type** | **Entrapment specific?** |  | **Prevalence finding** | **Timescale from event** |
| **ADULTS** | | | | | | | |
| Casacchia, M., Bianchini, V., Mazza, M., Pollice, R. & Roncone, R. | 2013 | Acute Stress Reactions and Associated Factors in the Help-Seekers after the L’Aquila Earthquake. | Single Study | No but entrapment was the strongest predictor of ASD in this cohort. | Earthquake | 4.9% met DSM-IV diagnostic criteria for ASD, 39.3% showed at least one symptom from each of the DSM-5 criterion for ASD  65.6% displayed ‘high levels of distress’ as measured on GHQ-12 | 1 month |
| **CHILDREN/ADOLESCENTS** | | | | | | | |
|  |  |  |  |  |  |  |  |
| ****No relevant articles identified**** | | | | | | | |
|  |  |  |  |  |  |  |  |

| ***Appendix 5 - Table 5.3 Summary of papers reporting Depression prevalence following non-MVC events where entrapment was common or likely*** | | | | | | | |
| --- | --- | --- | --- | --- | --- | --- | --- |
| **Author** | **Year of Publication** | **Title** | **Article Type** | **Entrapment specific?** |  | **Prevalence finding** | **Timescale from event** |
| **ADULTS** | | | | | | | |
| Aziz, S. & Aslam, N. | 2012 | Psychiatric Morbidity and Work and Social Adjustment Among Earthquake Survivors Extricated from under the Rubble. | Single Study | Yes | Earthquake | 17.5% | Timescale not stated |
| Cerdá, M. et al. | 2013 | PSYCHOPATHOLOGY IN THE AFTERMATH OF THE HAITI EARTHQUAKE: A POPULATION‐BASED STUDY OF POSTTRAUMATIC STRESS DISORDER AND MAJOR DEPRESSION. | Single Study | No but entrapment identified as significant risk factor in women but not men | Earthquake | 28.3% | 2 months-4 months |
| Tang, B., Liu, X., Liu, Y., Xue, C. & Zhang, L. | 2014 | A meta-analysis of risk factors for depression in adults and children after natural disasters. | Meta-Analysis | No but entrapment identified as significant risk factor in children but not in adults. | Multiple studies, natural disasters: (earthquakes, n=12, hurricane/tornadoes (n=6), tsunamis (n=1), floods (n=1), | 5.8%-54% | 6 weeks - 4 years (mean 11.03 months) |
| Petrucci, E. et al. | 2023 | Health Status Perception and Psychological Sequelae in Buried Victims: An Observational Study on Survivors of the Earthquake in Amatrice (Italy), Three Years Later. | Single Study | Yes | Earthquake | 57% | 3 years |
| **CHILDREN/ADOLESCENTS** | | | | | | | |
| Eksi, A. & Braun, K. L. | 2009 | Over-time changes in PTSD and depression among children surviving the 1999 Istanbul earthquake. | Single Study | No but “Only one variable - having been in serious personal danger (e.g., trapped in the house or under rubble) - was significantly associated with being symptomatic at follow-up” | Earthquake | 30.6% of children/adolescents met diagnostic thresholds for depression (all with comorbid PTSD) at baseline (1-2 months post event)  5.4% met diagnostic criteria for depression (half remained comorbid w/PTSD) at follow-up, 18-20 months post event). | 1 month-2 months, follow up 18 months-20 months |
| Tang, B., Liu, X., Liu, Y., Xue, C. & Zhang, L. | 2014 | A meta-analysis of risk factors for depression in adults and children after natural disasters. | Meta-Analysis | No but entrapment identified as significant risk factor in children but not adults | 11 studies, natural disasters: (earthquake (n=9), tornado (n=1), tsunami (n=1) | 7.5%-44.8% prevalence of depression in children | 1 month-3 years (mean 9.69 months) |
| Sharma, A. & Kar, N. | 2019 | Posttraumatic Stress, Depression, and Coping Following the 2015 Nepal Earthquake: A Study on Adolescents. | Single Study | No but entrapment identified as significant risk factor for PTSD, but not depression | Earthquake | 38.1% | 1 year |
| Marthoenis, M., Ilyas, A., Sofyan, H. & Schouler-Ocak, M. | 2019 | Prevalence, comorbidity and predictors of post-traumatic stress disorder, depression, and anxiety in adolescents following an earthquake. | Single Study | No. The injury of close family members and feeling stressed after the earthquake were statistically significant risk factors for depression. | Earthquake | 16.8% | 6 months |
| Qi, J., Yang, X., Tan, R., Wu, X. & Zhou, X. | 2020 | Prevalence and predictors of posttraumatic stress disorder and depression among adolescents over 1 year after the Jiuzhaigou earthquake. | Single Study | No but entrapment identified as significant risk factor | Earthquake | 64.5% consistent with depression  39.2% comorbid PTSD and depression | over 1 year |

| ***Appendix 5 - Table 5.4. Summary of papers reporting Anxiety prevalence following non-MVC events where entrapment was common or likely*** | | | | | | | |
| --- | --- | --- | --- | --- | --- | --- | --- |
| **Author** | **Year of Publication** | **Title** | **Article Type** | **Entrapment specific?** |  | **Prevalence finding** | **Timescale from event** |
| **ADULTS** | | | | | | | |
| Aziz, S. & Aslam, N. | 2012 | Psychiatric Morbidity and Work and Social Adjustment Among Earthquake Survivors Extricated from under the Rubble. | Single Study | Yes | Earthquake | ‘Anxiety-related disorders’;  Agoraphobia (25%)  Panic disorder (20%) | Timeframe not stated |
| Petrucci, E. et al. | 2023 | Health Status Perception and Psychological Sequelae in Buried Victims: An Observational Study on Survivors of the Earthquake in Amatrice (Italy), Three Years Later. | Single Study | Yes | Earthquake | 35% | 3 years |
| **CHILDREN/ADOLESCENTS** | | | | | | | |
| Marthoenis, M., Ilyas, A., Sofyan, H. & Schouler-Ocak, M. | 2019 | Prevalence, comorbidity and predictors of post-traumatic stress disorder, depression, and anxiety in adolescents following an earthquake. | Single Study | No, but injury, house destruction, fear of being in a building and gender were significant predictors for generalized anxiety disorder. | Earthquake | 32.1% | 6 months |

**
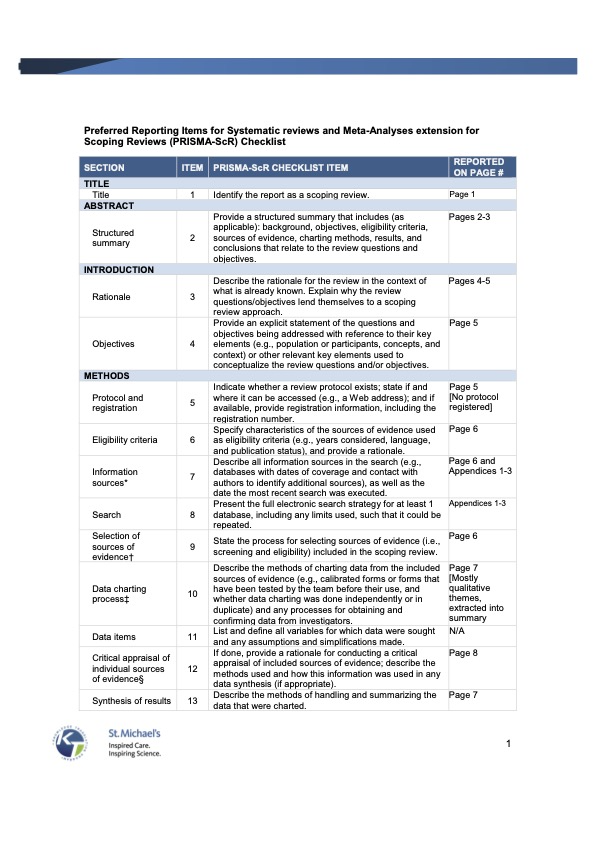
Appendix 6: PRISMA-ScR reporting checklist**


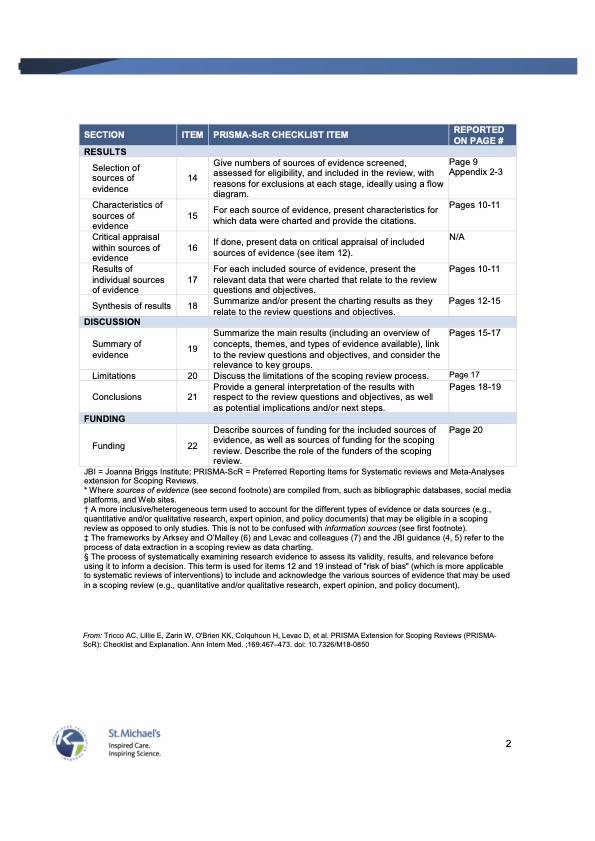


1. Full list of databases searched via NHS Knowledge and Library Hub available at <https://support.library.hee.nhs.uk/support/solutions/articles/27000028439-What-resources-are-searched-through-the-Knowledge-and-Library-Hub->
   AMBER: the home of ambulance services research (ir01808a) - National repository for Ambulatory care; Academic Search Index (asx) - core index prepared by EBSCO for discovery purposes; APA PsycArticles (edspdh) - full text collection; APA PsycBooks (edspzh); Aphasiology Archive (edsupa) - full text collection; BioOne Complete (edsbio) - STEM database; BMJ Best Practice (edsbmj) - National point of care tool; British Library EThOS (edsble) - Theses and dissertations; Center for Research Libraries (edscrl) - broad international database; CINAHL (cin20) - major literature database; ClinicalTrials.gov (edsclt) - details of clinical trials in progress and completed; Cochrane Database of Systematic Reviews (edschh); Complementary Index (edb) - core index prepared by EBSCO for discovery purposes; Digital Access to Scholarship at Harvard (DASH) (edshld) publications from a global university; Directory of Open Access Books (edsdob) - includes direct links to fulltext; Directory of Open Access Journals (edsdoj) - supports users using the full text limiter in the Hub to see more materials; eBook Collection (EBSCOHost) (nlebk) - EBSCO's ebook collection; including books from Gobi; Emerald Insight (edsemr) - details of journals from this publisher; Gale Health and Wellness (edsghw) - consumer health focus; Gale OneFile: Health and Medicine (edsgih) - wide range of health articles and news; GreenFILE (8gh) - environmental issues; International Clinical Trials Registry Platform ICTRP (edsict) - global clinical trials details - optional - log a Service request to add this as it requires an additional link; Journals@OVID (edsovi); Library, Information Science & Technology Abstracts (lxh); McGraw-Hill Medical (edsmgh) - medical textbooks; MEDLINE (cmedm) - major literature database; Minority Health Archive (edsuph) - minority health and health disparities research and policy; NICE Guidance (edsngc); NCI (National Cancer Institute at the National Institutes of Health) (edspdq) - full text collection; Oxford Bibliographies (edsobb); Oxford Clinical Psychology (edsocp); Oxford Handbooks Online (edsoho); Oxford Medicine Online (edsmeo) - please note that this has been deselected for the Hub instances in which the SWIMS library catalogue has been integrated; POLICY database from Analysis & Policy Observatory (edsapo) - health policy mostly non UK but includes similar health systems; PsychiatryOnline (edspsy) - psychiatry journals; Psychology and Behavioral Sciences Collection (pbh) - full text collection; Research Starters (ers) - panels with basic introductions on some topics; Royal Marsden Manual of Clinical Procedures (edsrmm) - chapter level indexing of RMM; ScienceDirect (edselp) - publisher database; SpringerProtocols (edsspo) - biomedical and life sciences protocols; Supplemental Index (edo) - core index prepared by EBSCO for discovery purposes; University Press Scholarship Online (edsups) - global academic book database; Wiley online reference works (edsmrw) - chapter level indexing of reference books; West Midlands Evidence Repository (WMER) (ir02173a) - regional NHS institutional repository [↑](#footnote-ref-1)
2. Full list of databases searched via NHS Knowledge and Library Hub available at<https://support.library.hee.nhs.uk/support/solutions/articles/27000028439-What-resources-are-searched-through-the-Knowledge-and-Library-Hub->

   AMBER: the home of ambulance services research (ir01808a) - National repository for Ambulatory care; Academic Search Index (asx) - core index prepared by EBSCO for discovery purposes; APA PsycArticles (edspdh) - full text collection; APA PsycBooks (edspzh); Aphasiology Archive (edsupa) - full text collection; BioOne Complete (edsbio) - STEM database; BMJ Best Practice (edsbmj) - National point of care tool; British Library EThOS (edsble) - Theses and dissertations; Center for Research Libraries (edscrl) - broad international database; CINAHL (cin20) - major literature database; ClinicalTrials.gov (edsclt) - details of clinical trials in progress and completed; Cochrane Database of Systematic Reviews (edschh); Complementary Index (edb) - core index prepared by EBSCO for discovery purposes; Digital Access to Scholarship at Harvard (DASH) (edshld) publications from a global university; Directory of Open Access Books (edsdob) - includes direct links to fulltext; Directory of Open Access Journals (edsdoj) - supports users using the full text limiter in the Hub to see more materials; eBook Collection (EBSCOHost) (nlebk) - EBSCO's ebook collection; including books from Gobi; Emerald Insight (edsemr) - details of journals from this publisher; Gale Health and Wellness (edsghw) - consumer health focus; Gale OneFile: Health and Medicine (edsgih) - wide range of health articles and news; GreenFILE (8gh) - environmental issues; International Clinical Trials Registry Platform ICTRP (edsict) - global clinical trials details - optional - log a Service request to add this as it requires an additional link; Journals@OVID (edsovi); Library, Information Science & Technology Abstracts (lxh); McGraw-Hill Medical (edsmgh) - medical textbooks; MEDLINE (cmedm) - major literature database; Minority Health Archive (edsuph) - minority health and health disparities research and policy; NICE Guidance (edsngc); NCI (National Cancer Institute at the National Institutes of Health) (edspdq) - full text collection; Oxford Bibliographies (edsobb); Oxford Clinical Psychology (edsocp); Oxford Handbooks Online (edsoho); Oxford Medicine Online (edsmeo) - please note that this has been deselected for the Hub instances in which the SWIMS library catalogue has been integrated; POLICY database from Analysis & Policy Observatory (edsapo) - health policy mostly non UK but includes similar health systems; PsychiatryOnline (edspsy) - psychiatry journals; Psychology and Behavioral Sciences Collection (pbh) - full text collection; Research Starters (ers) - panels with basic introductions on some topics; Royal Marsden Manual of Clinical Procedures (edsrmm) - chapter level indexing of RMM; ScienceDirect (edselp) - publisher database; SpringerProtocols (edsspo) - biomedical and life sciences protocols; Supplemental Index (edo) - core index prepared by EBSCO for discovery purposes; University Press Scholarship Online (edsups) - global academic book database; Wiley online reference works (edsmrw) - chapter level indexing of reference books; West Midlands Evidence Repository (WMER) (ir02173a) - regional NHS institutional repository [↑](#footnote-ref-2)
3. Full list of databases searched via NHS Knowledge and Library Hub available at<https://support.library.hee.nhs.uk/support/solutions/articles/27000028439-What-resources-are-searched-through-the-Knowledge-and-Library-Hub->

   AMBER: the home of ambulance services research (ir01808a) - National repository for Ambulatory care; Academic Search Index (asx) - core index prepared by EBSCO for discovery purposes; APA PsycArticles (edspdh) - full text collection; APA PsycBooks (edspzh); Aphasiology Archive (edsupa) - full text collection; BioOne Complete (edsbio) - STEM database; BMJ Best Practice (edsbmj) - National point of care tool; British Library EThOS (edsble) - Theses and dissertations; Center for Research Libraries (edscrl) - broad international database; CINAHL (cin20) - major literature database; ClinicalTrials.gov (edsclt) - details of clinical trials in progress and completed; Cochrane Database of Systematic Reviews (edschh); Complementary Index (edb) - core index prepared by EBSCO for discovery purposes; Digital Access to Scholarship at Harvard (DASH) (edshld) publications from a global university; Directory of Open Access Books (edsdob) - includes direct links to fulltext; Directory of Open Access Journals (edsdoj) - supports users using the full text limiter in the Hub to see more materials; eBook Collection (EBSCOHost) (nlebk) - EBSCO's ebook collection; including books from Gobi; Emerald Insight (edsemr) - details of journals from this publisher; Gale Health and Wellness (edsghw) - consumer health focus; Gale OneFile: Health and Medicine (edsgih) - wide range of health articles and news; GreenFILE (8gh) - environmental issues; International Clinical Trials Registry Platform ICTRP (edsict) - global clinical trials details - optional - log a Service request to add this as it requires an additional link; Journals@OVID (edsovi); Library, Information Science & Technology Abstracts (lxh); McGraw-Hill Medical (edsmgh) - medical textbooks; MEDLINE (cmedm) - major literature database; Minority Health Archive (edsuph) - minority health and health disparities research and policy; NICE Guidance (edsngc); NCI (National Cancer Institute at the National Institutes of Health) (edspdq) - full text collection; Oxford Bibliographies (edsobb); Oxford Clinical Psychology (edsocp); Oxford Handbooks Online (edsoho); Oxford Medicine Online (edsmeo) - please note that this has been deselected for the Hub instances in which the SWIMS library catalogue has been integrated; POLICY database from Analysis & Policy Observatory (edsapo) - health policy mostly non UK but includes similar health systems; PsychiatryOnline (edspsy) - psychiatry journals; Psychology and Behavioral Sciences Collection (pbh) - full text collection; Research Starters (ers) - panels with basic introductions on some topics; Royal Marsden Manual of Clinical Procedures (edsrmm) - chapter level indexing of RMM; ScienceDirect (edselp) - publisher database; SpringerProtocols (edsspo) - biomedical and life sciences protocols; Supplemental Index (edo) - core index prepared by EBSCO for discovery purposes; University Press Scholarship Online (edsups) - global academic book database; Wiley online reference works (edsmrw) - chapter level indexing of reference books; West Midlands Evidence Repository (WMER) (ir02173a) - regional NHS institutional repository [↑](#footnote-ref-3)
